# Design and evaluation of an augmented reality simulator using leap motion

**DOI:** 10.1049/htl.2017.0070

**Published:** 2017-09-14

**Authors:** Trinette Wright, Sandrine de Ribaupierre, Roy Eagleson

**Affiliations:** 1 Department of Biomedical Engineering, Western University, London, Canada N6A 3K7; 2 Department of Clinical Neurological Sciences, Western University, London, Canada N6A 3K7; 3 Department of Electrical and Computer Engineering, Western University, London, Canada N6A 3K7

**Keywords:** augmented reality, endoscopes, biomedical optical imaging, surgery, medical computing, leap motion, augmented reality simulator design, augmented reality simulator evaluation, medical field, surgical simulation, endoscopic third ventriculostomy, NeuroTouch, ETV simulator, training effectiveness, Unity, Vuforia, AR environment, virtual hand, trajectory log hles

## Abstract

Advances in virtual and augmented reality (AR) are having an impact on the medical field in areas such as surgical simulation. Improvements to surgical simulation will provide students and residents with additional training and evaluation methods. This is particularly important for procedures such as the endoscopic third ventriculostomy (ETV), which residents perform regularly. Simulators such as NeuroTouch, have been designed to aid in training associated with this procedure. The authors have designed an affordable and easily accessible ETV simulator, and compare it with the existing NeuroTouch for its usability and training effectiveness. This simulator was developed using Unity, Vuforia and the leap motion (LM) for an AR environment. The participants, 16 novices and two expert neurosurgeons, were asked to complete 40 targeting tasks. Participants used the NeuroTouch tool or a virtual hand controlled by the LM to select the position and orientation for these tasks. The length of time to complete each task was recorded and the trajectory log files were used to calculate performance. The resulting data from the novices' and experts' speed and accuracy are compared, and they discuss the objective performance of training in terms of the speed and accuracy of targeting accuracy for each system.

## 1 Introduction

Medical education and training are areas currently impacted by advances in virtual and augmented reality (VR and AR) [1–3]. Medical procedures are becoming increasingly more complex, so the use of VR and AR may prove beneficial by providing medical students and residents with more training opportunities [3, 4]. Endoscopic third ventriculostomy is a common neurological procedure that residents perform [5, 6]. The procedure is usually performed at the patient's bedside and is done without guidance from medical imaging. The correct placement of the catheter into the ventricle is important in relieving pressure and preventing any permanent damage [3, 6]. Many simulators have been designed and one such commercially available VR simulator that has been designed to practise this type of procedure is NeuroTouch [7]. NeuroTouch was developed by the National Research Council of Canada in partnership with over 20 research hospital across Canada [7]. This type of simulator combines graphics with a mechanical arm to simulate various types of procedures [7]. Unfortunately, these types of systems are very expensive, which limits the number of institutions that can provide these systems to students due to financial constraints [8].

A low cost, easily accessible ventriculostomy simulator was designed using an AR environment and the leap motion (LM) hand controller. This system has been deployed on a mobile platform, Android specifically. It will be evaluated in comparison with the NeuroTouch simulator using the same AR environment. The simulator has been tested using 18 participants in total with 16 novice users and two expert neurosurgeons. All participants completed 40 targeting tasks using both systems. The novice participants’ task completion times and accuracy in targeting have been compared against the expert to evaluate the usability of this AR system.

## 2 Methods

**Fig. 1:**
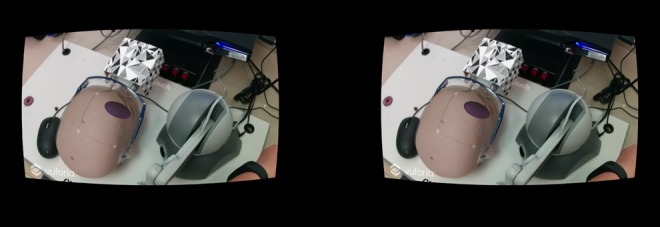
Application has the view from the camera displayed twice side-by-side to create a stereoscopic view to the user when viewed through the Google Cardboard 3D glass

This simulator was designed as an AR environment since as it allows the user to see the real world with virtual objects overlaid [9]. This type of environment was also chosen, as this system will be compared with physical simulator. The AR environment was created using Unity version 5.2.2f. Unity is a popular program that has been created for the design and development of video games [10, 11]. It allows for development of programs across multiple platforms such as Windows, iOS and Android [10, 11]. The most common display for VR and AR systems uses a head mounted display (HMD). There are many commercially available HMDs; however, due to improvements in hardware and graphics, it was decided the platform for this simulator would be a mobile base [12]. This provided greater accessibility for users. Smartphones contain sensors such as gyroscopes and have embedded cameras that can provide an immersive and interactive display for an AR environment [8, 12]. The LG Nexus 5 Android smartphone was specifically selected for this project. It was chosen because of its availability, developer device designation, and it is very affordable and fulfils the hardware specifications supplied by LM for running the device on a smartphone. The smartphone was paired with a set of Google Cardboard three-dimensional (3D) glasses to provide the user with stereoscopic view of the AR environment (Fig. 1). This is achieved by duplicating the camera view and placing them side-by-side on the screen.

**Fig. 2:**
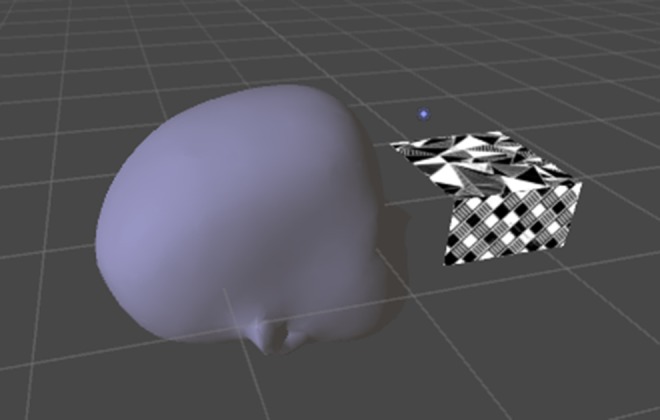
Virtual head loaded into Unity, showing the relative position of the Vuforia tracking marker

It was determined that image tracking should be used, as a virtual mannequin head could be more easily overlaid with the physical head used in NeuroTouch. Vuforia, version 6.2, was used as the image tracking software and was selected because it can be directly integrated with Unity. Vuforia is a stable image tracking platform that offers several types of image tracking and a 3D, multi-image cube was chosen as this offered users more freedom to move about with minimal tracking loss [13]. The image tracking is performed with the smartphone camera. Vuforia calculates the distance between the image and the camera and the orientation of the image. This is used to overlay virtual objects in a scene [14]. When the images used for tracking are detected, any virtual objects connected with this image are displayed. If the tracking of these images is lost, then the virtual objects will disappear. The multi-image cube is directly integrated with Unity (Fig. 2).

**Fig. 3:**
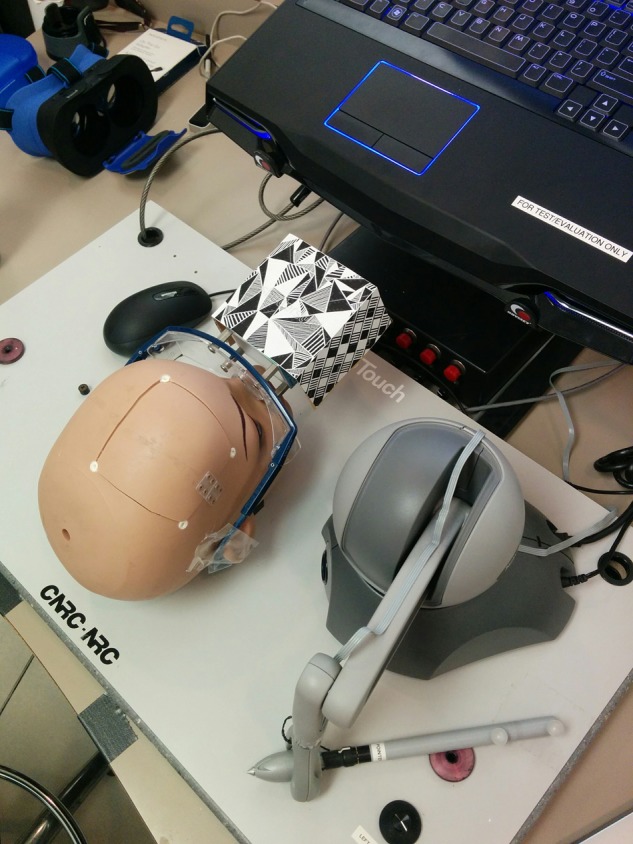
NeuroTouch haptic interface, mannequin head, and Vuforia image tracking cube. These are the physical input devices

The NeuroTouch surgical simulator consists of a physical mannequin head, haptic arm, and a foot pedal [1]. Accurate position and orientation tracking of the tip of the device is done by the arm. The cube was attached to a pair of safety glasses, which were then placed on the physical head (Fig. 3). The images were attached this way as it did not interfere with the operation of the mechanical arm [1].

**Fig. 4:**
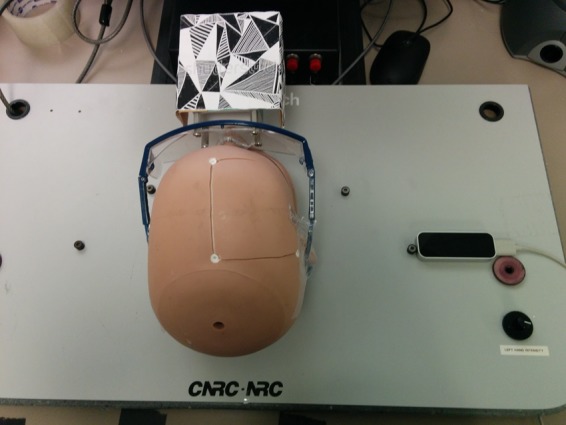
LM hand controller detects the movement and hand position of the user’s hands when held above the sensor

The LM is a 3D hand controller that uses two infrared cameras and three infrared sensors to detect the movement and position of the user's hands while held above the controller [15, 16]. The LM has a fingertip position accuracy of 0.01 mm [16]. The controller was directly integrated with Unity and was used to interact with the virtual objects within the environment. The LM can be directly connected to an Android smartphone, so no other hardware is required to run the simulator. Our laboratory is an alpha tester for the LM Android platform. The public does not have access to this application at the time of writing. The controller was placed beside the mannequin head instead of mounting it on the Google Cardboard glasses, so that the user's hands could not occlude the image mounted on the physical head which would cause tracking loss (Fig. 4).

**Fig. 5:**
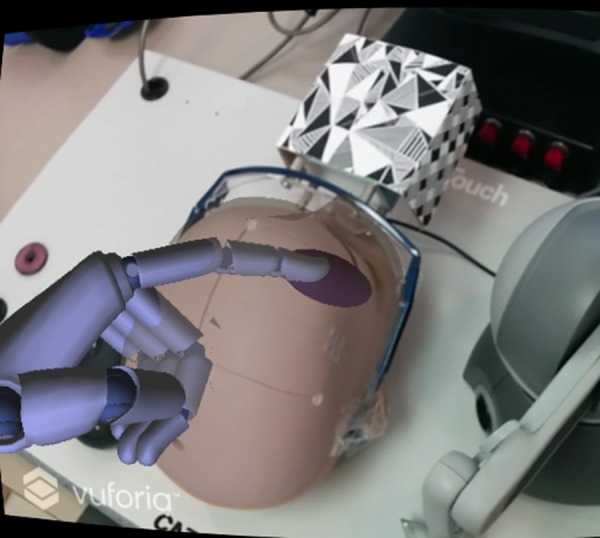
Virtual hand in the augmented environment show the LM representation of the tracked user pointing gesture

It was decided to use virtual hands to interact with the virtual objects. Using virtual hands instead of the user's physical hands for interaction with virtual objects was advantageous as the user could then explore the space within the virtual head. This exploration would not be possible with the user's physical hands. A set of virtual hands were incorporated into the environment and were created by LM and mimic the behaviour of the user's hands above the sensor (Fig. 5).

The participants were instructed to use the index finger of their dominant hand as the tool. The index finger was selected as it would be the most intuitive for the participants. The position of the tip of the finger and orientation of this finger were recorded.

**Fig. 6:**
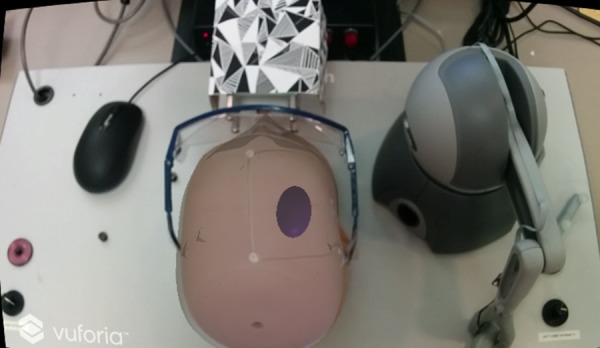
Each virtual ellipsoid is a target with random 3D position, size, and orientation in 3D, used to provide a well-posed targeting task

The 40 targeting tasks that were designed consisted of four ellipsoid practise targeting tasks and 36 ventricle targeting tasks. The practise ellipsoid targeting tasks were completed at the beginning to train the users on how to use the two different systems. The ellipsoids were random in size, position, and orientation within the virtual head (Fig. 6). The participants were instructed to use the NeuroTouch tooltip or their dominant hand virtual index finger to select the longest axis through each ellipsoid. Once the participant was confident, they had the correct placement and orientation, they would step on the NeuroTouch right foot pedal to record the tooltip position and orientation or press the action button on the Google Cardboard glasses to record the virtual fingertip position and orientation.

**Fig. 7:**
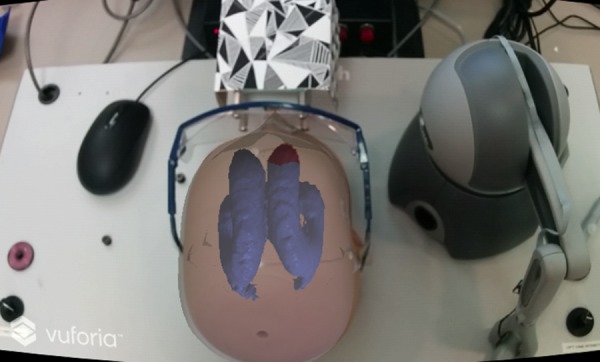
In the trials based on neuroanatomical structures, the lateral ventricles have the right anterior horn target highlighted in red

The ventricle targeting tasks consisted of placing the NeuroTouch tool arm or the virtual index finger, so that the angle of trajectory would go through the right anterior horn of the lateral ventricle (Fig. 7). The right anterior horn was selected as the NeuroTouch has a limited targeting range and can only target the right-hand side of the physical head. As novice users were selected as participants, the right anterior horn was highlighted, so that participants’ performance would not be impacted by their knowledge of anatomy. The ventricles were segmented from nine *t*1 weighted patient magnetic resonance imaging scans. These ventricles were then mirrored, adding extra trials as the left-hand side is not targeted. The participants were asked to complete the 18 ventricle targeting tasks twice, allowing for a check of practise effects.

**Fig. 8:**
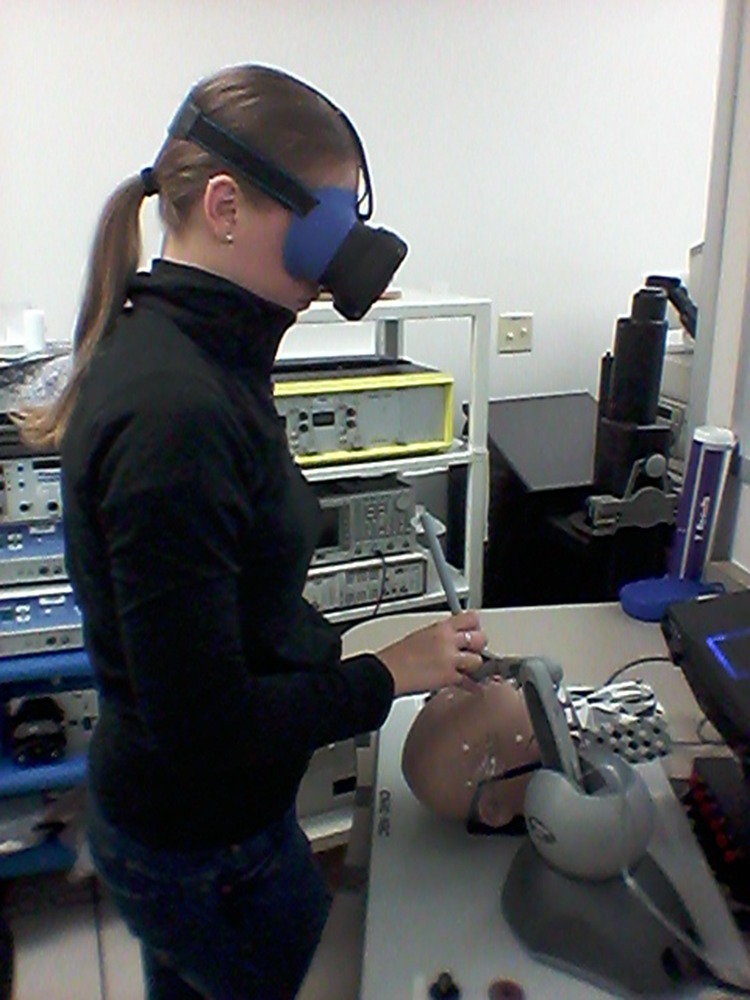
Participant using the NeuroTouch system, in the process of targeting – moving toward the anterior horn of a ventricle

The setup for each simulator was similar. The participants were positioned so that the mannequin head was facing away from them. The complete setup of the NeuroTouch simulator can be seen in Fig. 8.

**Fig. 9:**
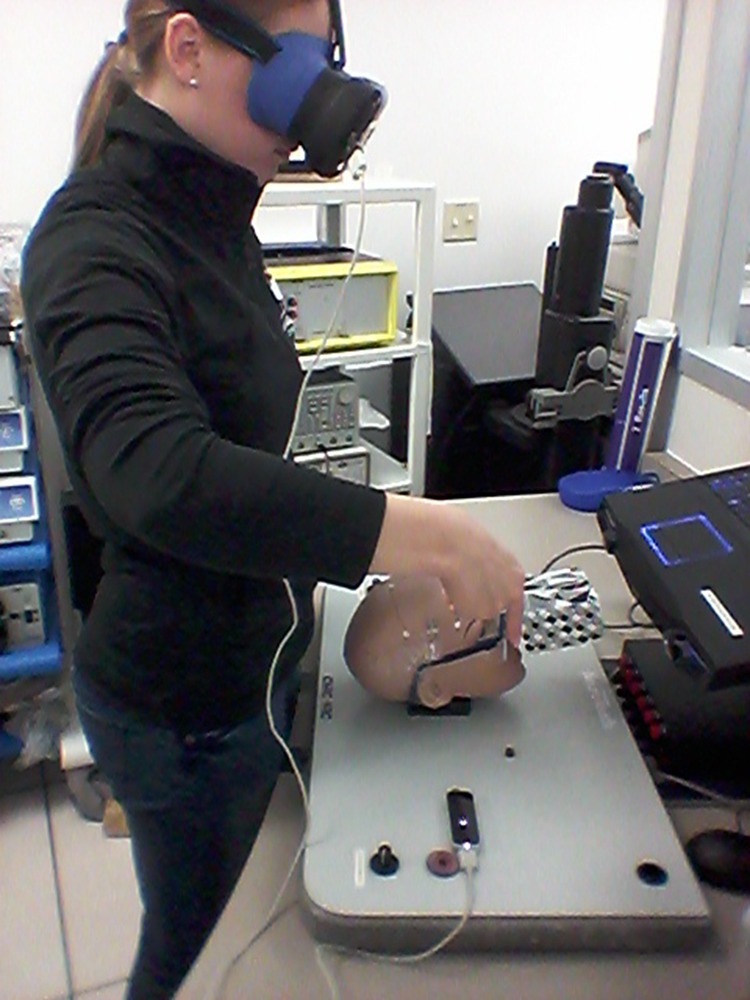
Participant using the NeuroTouch system, in the process of targeting – moving toward the anterior horn of a ventricle

The LM was placed on the right-hand side as well to mimic the setup of the NeuroTouch (Fig. 9).

The same 40 targeting tasks were used for both the NeuroTouch system and for the LM system. The NeuroTouch burr hole selection program was loaded for the users as it recorded the selected position and orientation of the tool arm when the right pedal was pressed. The participants were separated into two groups with one group starting with the NeuroTouch system and the other starting with the LM system. One expert started with the NeuroTouch system, whereas the other started with the LM system. The position and orientation of the smartphone glasses were recorded to assess how much the participants moved about in the environment. The completion time for each task was recorded.

The registration between the physical and virtual mannequin heads was used to transform the data collected from the Unity environment into the NeuroTouch space. This was done so the results from both systems could be directly compared.

## 3 Results

**Fig. 10:**
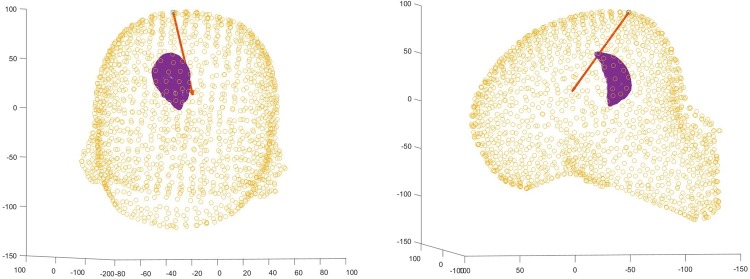
Front and side views of one user’s selected trajectory through the ventricle mesh using NeuroTouch

The task performance is the combined product of task speed and accuracy. For the tasks completed using NeuroTouch, this was extracted from the recorded trajectory logfiles (Fig. 10). The NeuroTouch records the position of the tip and orientation of the tool along with the time between the start of the task and the time when the participant presses the foot pedal.

**Fig. 11:**
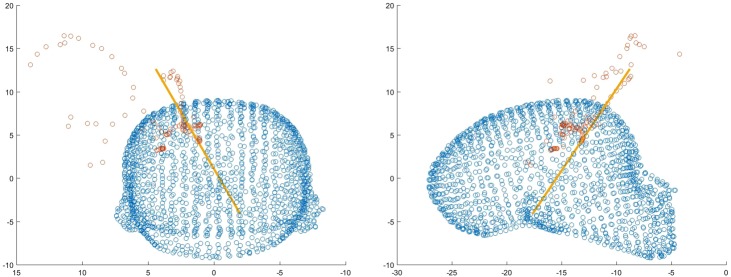
Front and side views of one user's index finger path with the final orientation of the finger displayed as a trajectory

This trajectory is also logged from the data collected from the Unity program. The Unity program provides more information than the NeuroTouch system as it records the entire approach of the participant to select the final position and trajectory (Fig. 11).

**Fig. 12:**
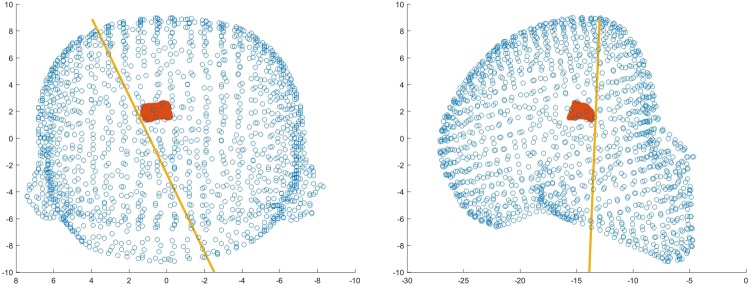
Front and side views of one user's selected trajectory through the ventricle mesh using LM

The final position and trajectory were selected for comparison with Neurotouch (Fig. 12).

**Fig. 13:**
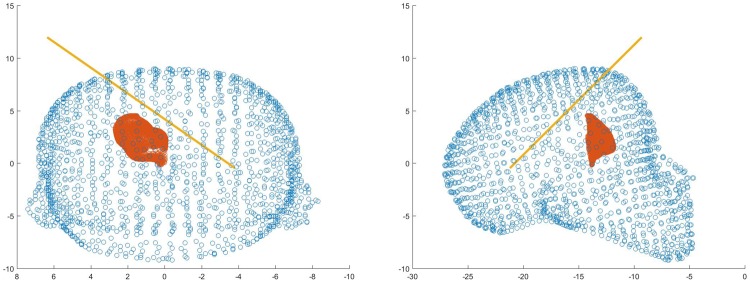
Front and side views of a novice users selected trajectory through the ventricle mesh using LM

**Fig. 14:**
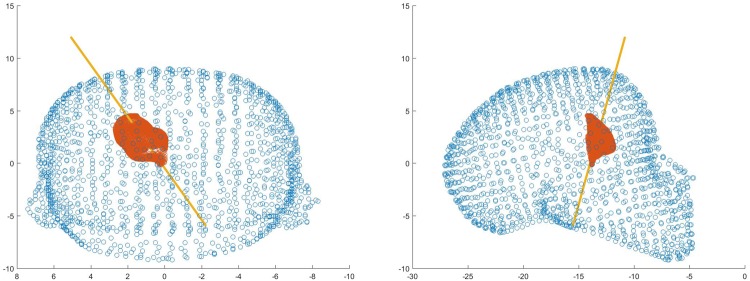
Front and side views of an expert users selected trajectory through the ventricle mesh using LM

Overall, all participants performed poorly using the NeuroTouch with both experts and novices on average missing the highlighted target. The participants’ performance improved for both the novice and expert users with the experts performing better than the novices (Figs. 13 and 14).

**Fig. 15:**
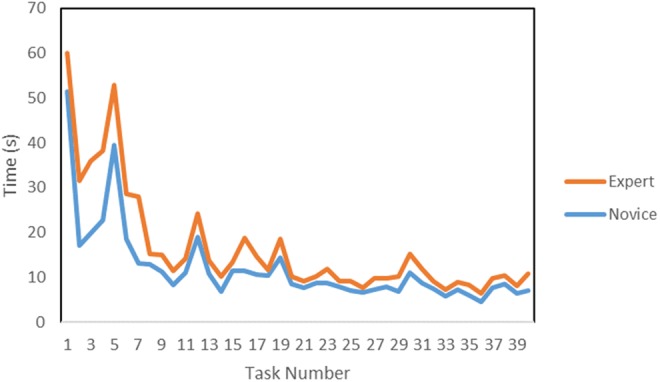
Average NeuroTouch novice and expert completion times for each task

The average task completion times of the novices and the experts were assessed as well as the overall trend in each participant's times and between participants. The average novice and expert task completion times for the NeuroTouch can be seen in Fig. 15.

**Fig. 16:**
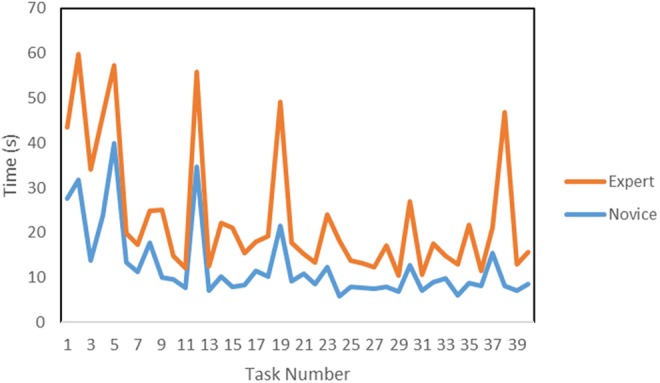
Average novice and expert task completion times using LM

The average novice and expert task completion times for the LM can be seen in Fig. 16.

Further usability of the system is provided by anecdotal feedback from the participants; a subjective questionnaire is also used to gather qualitative feedback, to complement our objective metrics.

## 4 Discussion

The preliminary results show some of the expected trends in the data. A training effect is observed and quantified across the datasets. As participants complete more trials, their task completion times decrease, at the same time as their accuracy improves. The ventricles were not placed in the correct anatomical position, so experts could not rely on their anatomical knowledge to correctly place the tools. This can be seen in the data as the experts and novices performed similarly when using the NeuroTouch. The experts and novices had similar task completion times which are consistent with the similar accuracies in targeting. There were some tracking issues with the NeuroTouch as some orientations of the tool arm would cause the image cube to be partially blocked and tracking would be lost. This leads to some longer completion times. The LM-based system provides a more intuitive 3D interactive experience than the stylus, though more technical refinements in the tracking robustness are needed, since from time to time it becomes inaccurate due to occlusions and lighting conditions. This can be seen in the participants’ task completion times. The experts did not complete the trials with the LM as quickly as the novices; however, the experts had a much higher targeting success rate than the novices. The full hand motion was recorded for the LM tasks that were not available with the NeuroTouch. This data can be used to assess the approach each user took to select the final position and orientation of the index finger and how far away from the centre the user was for each target.

## 5 Conclusion

We have created an affordable, easily accessible simulator that with further testing will become an intuitive and easy to use training and evaluation tool for surgical training. This system can be used to facilitate the targeting skills of clinicians and provide a system for planning procedures using patient datasets. The system makes use of AR/VR display modalities, which show promise for training and computer-assisted interventions [17]. The logfiles produced by this system can be used to assess the improvements in performance (in accord with methodologies developed previously [18–21]) and used to quantitatively assess the uptake of skills by the trainees. These Letters provide important insights into this application and should be considered by others who develop such systems [22, 23].
